# Effect of Walking Steps Measured by a Wearable Activity Tracker on Improving Components of Metabolic Syndrome: A Prospective Study

**DOI:** 10.3390/ijerph19095433

**Published:** 2022-04-29

**Authors:** Jae-Min Park, Ja-Eun Choi, Hye Sun Lee, Soyoung Jeon, Ji-Won Lee, Kyung-Won Hong

**Affiliations:** 1 Department of Family Medicine, Gangnam Severance Hospital, Yonsei University College of Medicine, 211 Eonju-ro, Gangnam-gu, Seoul 06273, Korea; milkcandy@yuhs.ac; 2 Department of Medicine, Graduate School of Medicine, Yonsei University, 50-1 Yonsei-ro, Seodaemun-gu, Seoul 03722, Korea; 3 Theragen Bio Co., Ltd., 145 Gwanggyo-ro, Suwon-si 16229, Korea; jaeun.choi@theragenbio.com; 4 Biostatistics Collaboration Unit, Yonsei University College of Medicine, 211 Eonju-ro, Gangnam-gu, Seoul 06273, Korea; hslee1@yuhs.ac (H.S.L.); jsy0331@yuhs.ac (S.J.); 5 Department of Family Medicine, Severance Hospital, Yonsei University College of Medicine, 50-1 Yonsei-ro, Seodaemun-gu, Seoul 03722, Korea

**Keywords:** metabolic syndrome, wearable activity tracker, walking, step count, physical activity

## Abstract

We compared the improvement in components of metabolic syndrome (MS) before and after lifestyle modification, as determined by daily step counts (on a wrist-worn Fitbit^®^) in participants with and without MS recruited from volunteers attending medical health checkup programs. A linear mixed model was used to analyze the change in MS components between participants with and without MS by group × time interaction. Multiple logistic regression analysis after adjustment for confounders was used to obtain odds ratios (ORs) and 95% confidence intervals (CIs) for improvements in MS components per 1000-steps/day increments. Waist circumference, triglycerides, fasting plasma glucose, and diastolic blood pressure were significantly different between participants with and without MS (group × time: *p* = 0.010, *p* < 0.001, *p* = 0.025, and *p* = 0.010, respectively). Multivariable-adjusted ORs (95% CI) of improvement in MS components per 1000-steps/day increments were 1.24 (1.01–1.53) in participants with and 1.14 (0.93–1.40) in participants without MS. Walking improved MS components more in individuals with than without MS. From a public health perspective, walking should be encouraged for high-risk MS individuals.

## 1. Introduction

Metabolic syndrome (MS), which is characterized by a clustering of metabolic abnormalities including central obesity, high triglycerides (TG), low high-density lipoprotein cholesterol (HDL-C), high blood sugar, and elevated blood pressure (BP), is thought to be linked to the development of numerous medical disorders [1]. Individuals with MS are more susceptible to infectious, neurodegenerative, cardiovascular, and cerebrovascular diseases, as well as certain cancers, which are the leading causes of mortality in adults worldwide [2,3,4,5,6]. MS has become a pandemic that threatens public health [7]. Therefore, preventing and managing MS is important from a public health perspective.

The pathogenesis of MS is believed to involve the interplay of genetic and environmental factors [8]. As environmental factors are controllable, appropriate control may contribute to the prevention and management of MS. Sedentary time is associated with MS and its components [9,10]. Thus, decreasing sedentary time and increasing physical activity are effective for preventing MS and the aggravation of its components.

Walking is a simple method to increase physical activity and is not restricted by place. Individuals who walk between 10,000 and 12,000 steps per day generally have a lower waist circumference, body fat percentage, and body mass index (BMI) [11,12,13]. Furthermore, Sisson et al. showed that individuals who walked more than 10,000 steps per day are likely to have a lower prevalence of MS [14]. However, few studies have investigated the effect of daily step count on improvements in MS components in participants with and without MS [15,16]. Identifying which group experiences a marked effect on MS from walking can facilitate a recommendation to encourage walking in this population.

The recent development of wearable activity trackers has facilitated easy monitoring and collection of lifelog data, such as daily steps. The advantages of wearable device technology include the possible increase in personal healthcare awareness and disease prevention. Recent surveys of worldwide fitness trends showed wearable technology as a top fitness trend [17,18,19]. However, few clinical trials have been conducted on their effectiveness in healthcare [20].

Therefore, this study was conducted to collect daily step counts using a wearable activity tracker, to compare the health status information before and after lifestyle modification, and to comparatively examine the improvement in MS components relative to daily step counts in participants with and without MS. Since few studies have collected complex lifelog data and assessed the quality of the data, we present data quality standards for wearable-device-based lifelog data research.

## 2. Materials and Methods

### 2.1. Study Design and Participants

In this study, participants were recruited from among volunteers who attended the medical health checkup programs at the Department of Family Medicine, Gangnam Severance Hospital, Seoul, Korea. Volunteers were able to participate regardless of the presence or absence of MS. Subsequently, participants were divided into those with and those without MS based upon anthropometric measurements and blood tests. Each participant wore on their wrist a Fitbit^®^ device, which is one of the most common wireless physical activity trackers in the consumer market and is known to be a reasonable and reliable tool for physicians to estimate the distance walked from the recorded step counts of patients [21]. All participants were instructed to wear their Fitbit^®^ device all day except when bathing or charging the device, and were encouraged to modify their lifestyle, such as walking more than they had prior to enrollment in the study.

Written informed consent was obtained from each participant prior to data collection. The study protocol was in accordance with the principles described in the 1975 Declaration of Helsinki, as confirmed a priori by the Institutional Review Board of Theragen Bio Institute, which approved this study (Institutional Review Board number: 700062-20190819-GP-006-02).

### 2.2. Data Preprocessing

**Figure 1:**
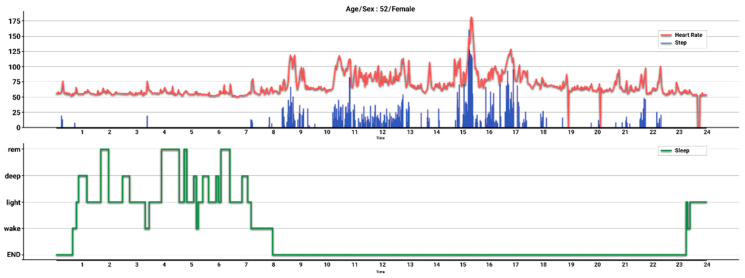
Steps, heart rate, and sleep cycle in a day, as collected by the Fitbit^®^ device, of a female participant aged 52 years. Schemes follow the same formatting. Indicated are steps (blue line), heart rate (red line), and sleep cycle (green line) with sleep/wake time and four stages of sleep: “wake,” “light,” “deep,” and “REM”.

The raw data of daily step count obtained using the 3-axis accelerometer of Fitbit^®^, which allows the device to determine the frequency, duration, intensity, and patterns of a participant’s movement, were uploaded to the Fitbit^®^ database [22,23]. The Fitbit^®^ device collects steps and heart rate in minus epochs, and measures sleep/wake time and sleep stages across four levels—“wake,” “light,” “deep,” and “REM” (Figure 1) [24]. The heart rate value corresponding to 0 in Figure 1 means that a user did not wear a device at that time. For the data analysis of step count, it was necessary to separate the meaning of the estimated step counts in accordance with the participants’ activity states, such as rest, and to assign a null value for heart rate when not wearing the device. With these considerations, we conducted a quality check to select the available data for inclusion in the final analysis, based on the following criteria for each participant: Criterion (1), duration of wearing the Fitbit^®^ device between baseline and follow-up; Criterion (2), average time wearing the device in a day; and Criterion (3), compliance, which we defined as the ratio of the day with overtime based on Criterion 2 in the intervention.

**Figure 2:**
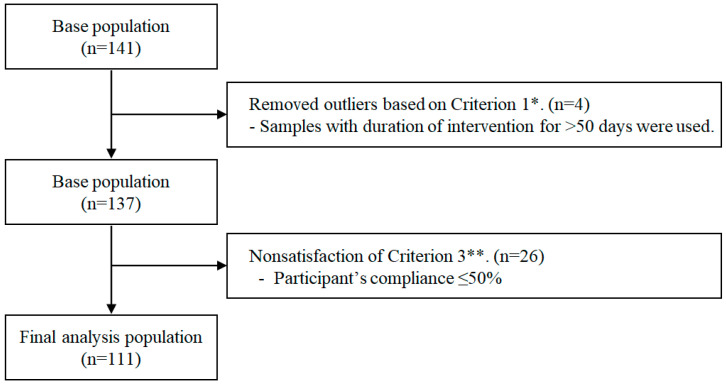
Flow chart of the selection of the study population. * Criterion 1 was defined as the duration of wearing Fitbit^®^ device between baseline and follow-up. ** Criterion 3 was defined as the participant’s compliance, calculated as the ratio of the day with overtime estimated based on the average duration that the device was worn per day during the intervention.

We excluded samples that constituted an outlier for Criterion 1 and did not satisfy Criteria 2 and 3. We determined a quarter value (Q1) for all participants’ average wear-time as Criterion 2. Based on this value, we calculated a participant’s compliance and determined a Q1 of compliance as Criterion 3. Finally, we defined the exclusion criterion as a 50% or lower ratio of days wearing a Fitbit^®^ device for more than 16 h (two-thirds of one day) between baseline and follow-up. Finally, 111 participants were included in the final analysis dataset. A flow chart of the selection process of the study population is depicted in Figure 2.

### 2.3. Measurement of Daily Step Count

Each participant had a different wear-time per day as well as a different duration between baseline and follow-up. Thus, the representative activity intensity of participants was required for the final analysis. We specified an average daily step count, calculated as the sum of the daily step count divided by the total duration (days) of the intervention that satisfied the criteria, as the normalized value of each participant’s activity.

### 2.4. Measurement of Anthropometric and Laboratory Data

Body weight and height were measured with the participants wearing light indoor clothing and no shoes, and BMI (kg/m^2^) was calculated. Systolic BP (SBP) and diastolic BP (DBP) measurements were obtained from the participants in a sitting position after a 10 min resting period. Blood samples were collected from patients following at least 8 h of fasting. TG, HDL-C, and fasting plasma glucose (FPG) were measured using the AU5822 automated clinical analyzer (Beckman Coulter, Brea, CA, USA).

### 2.5. Definition of Terms

We used the definitions of MS and its components according to the modified National Cholesterol Education Program Adult Treatment Panel III (NCEP-ATP III) ethnicity-specific guidelines [25] and the Korean Society for the Study of Obesity [26]. Specifically, we defined MS as the presence of three or more of the following components: (1) abdominal obesity (waist circumference ≥ 90 cm for males and ≥85 cm for females); (2) high TG (≥150 mg/dL); (3) low HDL-C (<50 mg/dL); (4) high FPG (≥100 mg/dL) and/or medication or insulin use for diabetes; and (5) elevated blood pressure (SBP/DBP ≥ 130/85 mmHg or use of antihypertensive medication). Participants with MS were defined as individuals having three or more MS components at baseline. Participants without MS were defined as individuals having less than three MS components at baseline.

An improvement in the number of MS components was defined as a decrease in the number of MS components after the intervention.

### 2.6. Statistical Analysis

Normal distribution was evaluated by the determination of skewness using the Kolmogorov–Smirnov test. TG values showed a skewed distribution. The characteristics of the participants according to sleep quality were expressed as the mean ± standard deviation, median (interquartile range), or percentages and were compared using the independent *t*-test or Wilcoxon rank-sum test for continuous variables and the chi-square test for categorical variables.

Baseline characteristics of participants and outcomes after lifestyle modification are presented as the mean ± standard deviation or median (interquartile range) for continuous variables or n (%) for categorical variables. Changes in continuous measures between baseline and post-intervention (the lifestyle modification) measurements were tested using the paired *t*-test or Wilcoxon signed-rank test for continuous variables.

A linear mixed model was used to analyze the change of MS components between participants with and without MS considering the group and time. A covariance pattern model was used in the linear mixed model. As a fixed effect, group, time, and group × time were considered. The *p*-values of group, time, and group × time were obtained, and the *p*-value of group × time indicated the difference of change between groups with and without MS for about 2 months. Results are presented using a linear mixed model and mean profile plots.

Multiple logistic regression analysis after adjustment for confounding factors including age, sex, smoking, and alcohol consumption was used to determine the odds ratios (ORs) and 95% confidence intervals (CIs) for the improvement in the number of MS components per 1000-steps/day increments. In this multiple logistic regression analysis, the main independent variable was step counts and the dependent variable was improvement in the number of MS components.

To determine the optimal cutoff points for improvement in the number of MS components, the receiver operating characteristic (ROC) curves of daily step count for the improvement in a number of MS components were drawn and the area under the curve (AUC) was calculated for participants with and without MS. To determine the optimal cutoff points, points on the ROC curve with the maximum Youden Index [sensitivity + specificity − 1] were calculated in participants with and without MS.

All statistical analyses were performed using SAS (ver. 9.4; SAS Institute, Inc., Cary, NC, USA) and R (ver. 4.0.2; R Foundation for Statistical Computing, Vienna, Austria). Statistical significance was set at *p*-value < 0.05.

## 3. Results

Table 1 presents the intervention period and compliance of participants. There was no intergroup difference in the intervention period, total step count per day, and compliance between participants with and without MS.

Table 2 shows the baseline characteristics of participants and outcomes after lifestyle modification. Waist circumference, TG, SBP, and DBP significantly decreased after lifestyle modification in participants with MS; however, only waist circumference significantly decreased in participants without MS.

**Figure 3:**
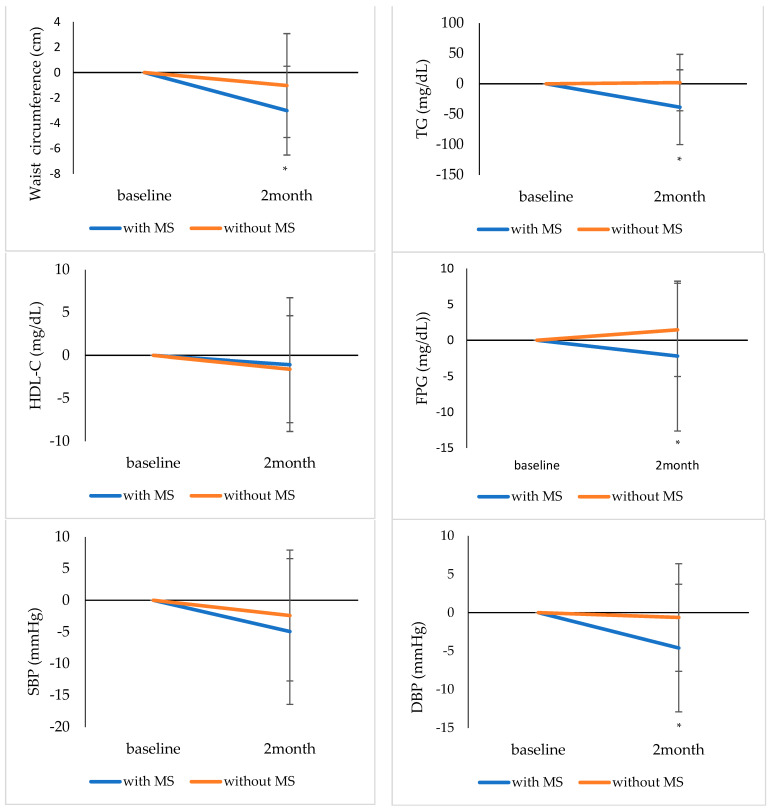
Mean profile plots of the components of MS by the presence of MS. The symbol * indicates that the group × time *p*-value < 0.05. MS, metabolic syndrome; TG, triglycerides; HDL-C, high-density lipoprotein cholesterol; FPG, fasting plasma glucose; SBP, systolic blood pressure; DBP, diastolic blood pressure.

Figure 3 shows mean profile plot obtained using a linear mixed model. Considering group and time, waist circumference, TG, FPG, and DPB were significantly different between participants with and without MS (group × time: *p* = 0.010, *p* < 0.001, *p* = 0.025, and *p* = 0.010, respectively); in contrast, HDL-C and SBP were not significantly different between participants with and without MS.

Table 3 presents the ORs for improvement in a number of MS components per 1000-steps/day, as determined using multiple logistic regression analysis. The multivariable-adjusted OR (95% CI) of the improvement in the number of MS components for a 1000-steps/day increment was 1.24 (1.01–1.53) in participants with MS and 1.14 (0.93–1.40) in participants without MS.

**Figure A1:**
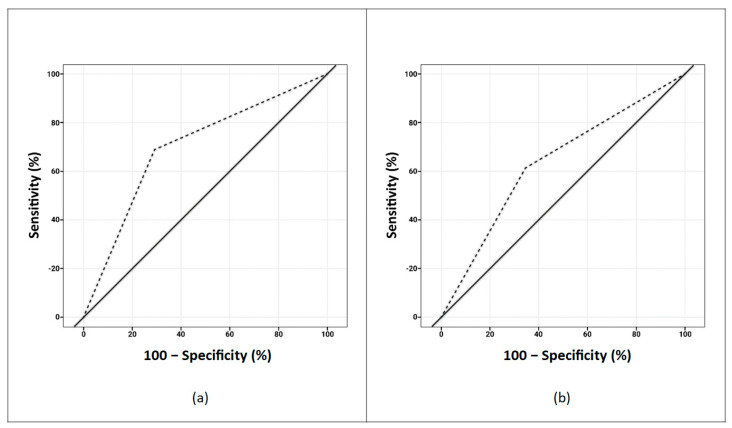
Receiver operating characteristic curves of daily step count required for improvement in the number of metabolic syndrome components in participants (**a**) with metabolic syndrome and (**b**) without metabolic syndrome. The daily step counts required to maximize the Youden Index in participants with and without metabolic syndrome were 11,438 (sensitivity 69.2%, specificity 70.6%) and 12,382 (sensitivity 61.5%, specificity 65.5%), respectively.

Figure A1 illustrates the ROC curves of daily step counts required for improvement in a number of MS components for participants (a) with MS and (b) without MS. The daily step count required to maximize the Youden Index in participants with and without MS was 11,438 (sensitivity 69.2%, specificity 70.6%) and 12,382 (sensitivity 61.5%, specificity 65.5%), respectively.

## 4. Discussion

In this study that analyzed the association between MS and the step count measured by Fitbit^®^, we found that the effect of walking on the improvement of MS components was greater in individuals with MS than in those without MS. Previous studies have shown that interventions to promote physical activity in individuals in the metabolic high-risk group are effective. Herzig et al. showed that walking, as an intervention, improved visceral fat area and insulin resistance in a Finnish population at high risk for type 2 diabetes [27]. Bhopal et al. also indicated that physical activity intervention resulted in significant weight loss in South Asian individuals who were at high risk of type 2 diabetes [28]. Our findings agree with the results of previous studies, which showed that interventions that promote physical activity improve metabolic parameters in the group at high risk for MS. In addition, this study suggests that walking should be encouraged in high-risk groups where MS poses a public health threat. We believe that this is the first study to reveal the effects of daily step counts measured by a wearable activity tracker on improving MS components in a comparative analysis of data from both participants with and without MS. Additionally, we suggest criteria for ensuring a quality check of the data collected on daily step counts, which are very complex.

We used the covariance pattern model, one of the linear mixed models, to compare the change of MS components between participants with and without MS by group × time interaction and found that there was a significant difference in some MS components, including TG, FPG, and DPB, between participants with and without MS. Therefore, we confirmed that the changes of the MS components due to walking were different between the two groups, and the association with the step counts of each of the two groups was verified.

In this study, the daily step counts required to improve the number of MS components were 11,438 and 12,382 in Korean individuals with and without MS, respectively. In a previous study, Bailey et al. reported that between 10,000 and 12,000 steps per day seems to be a reasonable recommendation that was associated with the lowest BMI and body fat composition in young adult women [12]. Furthermore, Krumm et al. demonstrated that postmenopausal women who took >10,000 steps per day had more favorable adiposity profiles [13]. Our findings agree with the results of the previous study that showed that people who walk between 10,000 and 12,000 steps per day generally have a lower waist circumference, body fat percentage, and BMI [11,12,13]. Moreover, our findings suggest that taking 11,400–12,400 steps per day could improve the number of MS components.

This study has several limitations that should be considered when interpreting the findings. First, this study analyzed a relatively small number of participants in each group. Further research using a larger sample size is needed. Second, as this was an exploratory study, we only applied the covariance pattern model from among the various linear mixed model methods. However, when performing further studies prospectively based on this study, it will be necessary to additionally apply the unconditional means model and unconditional growth model proposed by Singer [29]. Third, we did not match confounding characteristics between participants with MS and participants without MS, as participants were enrolled from among volunteers who attended medical health checkup programs. However, to minimize this limitation, we included significant covariates, including age, sex, smoking, and alcohol consumption that might potentially affect MS components as confounding variables in multiple logistic regression analysis. Further research that matches confounding characteristics is needed. Fourth, an improvement in the number of MS components was defined as a decrease in the number of MS components after the intervention. In particular, a ceiling effect may exist for the participants without MS. For this reason, we did not analyze the actual continuous values, but categorized them by logistic regression. Fifth, other covariates not included in the regression models might have altered the significance of the findings. Other covariates, such as dietary intake and socioeconomic status, could influence MS and its components. Several studies indicated that household income and education level are related to risk of MS in a sex-specific manner [30,31]. However, as this study did not collect these covariates, we could not include them in the regression model. Further research that includes other covariates in the regression model is needed to confirm the findings. Lastly, this study was confined to the Korean population and, thus, the findings may have limited generalizability with regard to other ethnic groups. Despite these potential limitations, we believe that our findings have clinical implications with regard to public health strategies for the prevention and management of MS. Furthermore, we suggest criteria for a quality check of data pertaining to the daily step counts, which are very complex.

## 5. Conclusions

In this study that analyzed the number of step counts measured by Fitbit^®^, the effect of walking on the improvement of MS components was greater in individuals with MS than in those without MS. This study suggests that, from a public health perspective, walking should be encouraged in the population at high risk for MS.

## Figures and Tables

**Table 1:** Intervention period, total step counts, and compliance of participants.

	Participants with Metabolic Syndrome (N = 43)	Participants without Metabolic Syndrome (N = 68)	*p*-Value
Intervention period (days)	59.6 ± 5.6	59.6 ± 5.7	0.982
Total step counts per day	11681 ± 3198	11651 ± 4227	0.968
Compliance (%)	85.8 ± 13.4	81.6 ± 15.3	0.140

Data are presented as the mean ± standard deviation. *p*-values were obtained using the paired *t*-test.

**Table 2:** Baseline characteristics of participants and outcomes after the lifestyle modification.

Variables	Participants with Metabolic Syndrome		*p*-Value	Participants without Metabolic Syndrome		*p*-Value
	(N = 43)			(N = 68)		
	Baseline	Post-Intervention		Baseline	Post-Intervention	
Age, years	59.4 ± 10.0			47.7 ± 14.1		
Male sex	8 (18.6)			12 (17.7)		
BMI, kg/m^2^	26.9 ± 2.1	26.3 ± 2.1	<0.001	23.8 ± 2.8	23.4 ± 2.5	<0.001
Waist circumference, cm	89.4 ± 4.9	86.3 ± 6.5	<0.001	80.4 ± 7.3	79.3 ± 6.7	0.043
TG, mg/dL	147.0 (106.0–185.0)	116.0 (84.0–147.0)	<0.001	82.5 (64.5–120.5)	81.5 (59.0–119.5)	0.807
HDL-C, mg/dL	54.4 ± 11.2	53.4 ± 9.4	0.376	63.2 ± 11.6	61.6 ± 10.2	0.038
FPG, mg/dL	107.4 ± 14.7	105.2 ± 12.6	0.175	94.7 ± 6.9	96.1 ± 8.1	0.070
SBP, mmHg	129.7 ± 10.2	124.8 ± 9.4	0.008	115.4 ± 10.8	113.1 ± 11.2	0.062
DBP, mmHg	84.4 ± 9.0	79.8 ± 8.0	0.001	72.3 ± 8.3	71.5 ± 8.3	0.469
Medication for hypertension	21 (48.8)			2 (2.9)		
Medication for diabetes	6 (14.0)			2 (2.9)		
Smoking	7 (16.3)			2 (2.9)		
Alcohol consumption	10 (23.3)			20 (29.4)		

Data are presented as the mean ± standard deviation or median (interquartile range) for continuous variables, or n (%) for categorical variables. *p*-values were obtained using the paired *t*-test or Wilcoxon signed-rank test, and represent the difference in each variable between baseline and post-intervention in each group. BMI, body mass index; TG, triglycerides; HDL-C, high-density lipoprotein cholesterol; FPG, fasting plasma glucose; SBP, systolic blood pressure; DBP, diastolic blood pressure.

**Table 3:** Improvement in the number of metabolic syndrome components per 1000-steps/day increments.

Analysis	Participants with Metabolic Syndrome, OR (95% CI)	Participants without Metabolic Syndrome, OR (95% CI)
Unadjusted (per 1000-steps/day)	1.30 (1.04–1.61)	1.13 (0.93–1.37)
Multivariable-adjusted * (per 1000-steps/day)	1.24 (1.01–1.53)	1.14 (0.93–1.40)

Data are presented as odds ratios (ORs) and 95% confidence intervals (CIs). * Adjusted for age, sex, smoking, and alcohol consumption.

## Data Availability

The data presented in this study are available on request from the corresponding author. The data are not publicly available due to ethical restrictions.
